# High-Voltage β-Ga_2_O_3_ Schottky Diode with Argon-Implanted Edge Termination

**DOI:** 10.1186/s11671-018-2849-y

**Published:** 2019-01-07

**Authors:** Yangyang Gao, Ang Li, Qian Feng, Zhuangzhuang Hu, Zhaoqing Feng, Ke Zhang, Xiaoli Lu, Chunfu Zhang, Hong Zhou, Wenxiang Mu, Zhitai Jia, Jincheng Zhang, Yue Hao

**Affiliations:** 1 State Key Discipline Laboratory of Wide Bandgap Semiconductor Technology, School of Microelectronics, Xidian University, Xi’an, 710071 China; 2 State Key Laboratory of Crystal Materials, Key laboratory of Functional Crystal Materials and Device, Shandong University, Jinan, 250100 China

**Keywords:** β-Ga_2_O_3_ Schottky diode, Argon implantation, Edge termination

## Abstract

The edge-terminated Au/Ni/β-Ga_2_O_3_ Schottky barrier diodes were fabricated by using argon implantation to form the high-resistivity layers at the periphery of the anode contacts. With the implantation energy of 50 keV and dose of 5 × 10^14^ cm^−2^ and 1 × 10^16^ cm^−2^, the reverse breakdown voltage increases from 209 to 252 and 451 V (the maximum up to 550 V) and the Baliga figure-of-merit (V_BR_^2^/R_on_) also increases from 25.7 to 30.2 and 61.6 MW cm^−2^, about 17.5% and 140% enhancement, respectively. According to the 2D simulation, the electric fields at the junction corner are smoothed out after argon implantation and the position of the maximum breakdown electric filed, 5.05 MV/cm, changes from the anode corner at the interface to the overlap corner just under the implantation region. The temperature dependence of the forward characteristics was also investigated.

## Background

Development of high-power devices using ultra-wide-bandgap semiconductor materials such as Ga_2_O_3_, AlN, diamond, etc. is accelerating in recent years. The bandgap of β-Ga_2_O_3_ is as large as 4.8–4.9 eV and the breakdown field of β-Ga_2_O_3_ is estimated to be 8 MV/cm, about three times larger than that of 4H-SiC and GaN. The Baliga’s figure of merit, 3400, is at least ten times larger than that of 4H-SiC and four times larger than that of GaN [1]. Furthermore, the large single crystal and low-cost β-Ga_2_O_3_ substrate can be fabricated with melt-growth methods such as floating-zone (FZ) [2] and edge-defined film-fed growth (EFG) [3, 4]. The electron density can be controlled over a wide range from 10^16^ to 10^19^ cm^−3^ by doping with Sn, Si, or Ge [5–7]. These excellent properties make β-Ga_2_O_3_ ideal for low loss, high-voltage switching and high-power applications, including high-breakdown voltage Schottky barrier diode (SBD) and metal-oxide-semiconductor field-effect transistor (MOSFET) [8–12]. Schottky barrier diodes possess the advantages of fast switching speed and low forward voltage drop in comparison with p-n junction diode, which can decrease the power loss and improve the efficiency of power supplies.

Although large breakdown voltages of 1016 V, 2300 V, and 1600 V have been obtained in β-Ga_2_O_3_ Schottky barrier diodes without edge termination, they are all about 34%, 8%, and 10% of the ideal value [10, 13, 14]. To relieve the electric field crowding effect and fully realize the voltage potential of β-Ga_2_O_3_, suitable edge terminations must be designed. There are a number of edge termination techniques to increase the device breakdown voltage such as field plates, floating metal rings, trench MOS structure, implanted guard rings, and junction termination extension (JTE) [12, 15–17]. However, implanted guard rings and JTE structure are not applicable to Ga_2_O_3_ Schottky diode due to the lack of p-type doping. H. Matsunami and B. J. Baliga put forward an edge termination structure, using argon implantation to form a high-resistivity amorphous layer at the edges of anode, to reduce the electric field crowding [18–22], which is a simple technique with no multi-photolithography or deep trench etching steps required, and it is widely used in SiC and GaN rectifiers to smooth out the electric field distribution around the rectifying contact periphery [15, 23, 24].

In this paper, the vertical edge-terminated β-Ga_2_O_3_ Schottky diodes were fabricated with argon implantation at the edges of Schottky contacts. The capacitance–voltage (C–V) and temperature-dependent current density-voltage (J-V) characteristics were recorded using Keithley 4200 semiconductor characterization system and the electric field distribution was also analyzed.

## Methods/Experimental

**Fig. 1:**
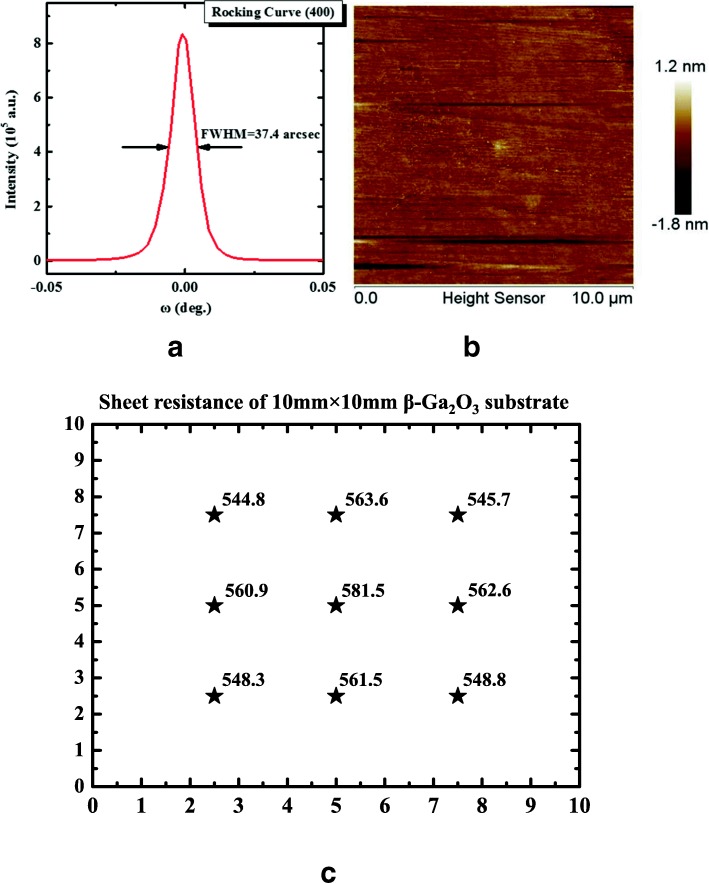
**a** XRD rocking curve and **b** AFM image of the β-Ga_2_O_3_ drift layer mechanically exfoliated from (100) β-Ga_2_O_3_ substrate **c** measured sheet resistance of 10 mm × 10 mm β-Ga_2_O_3_ substrate

**Fig. 2:**
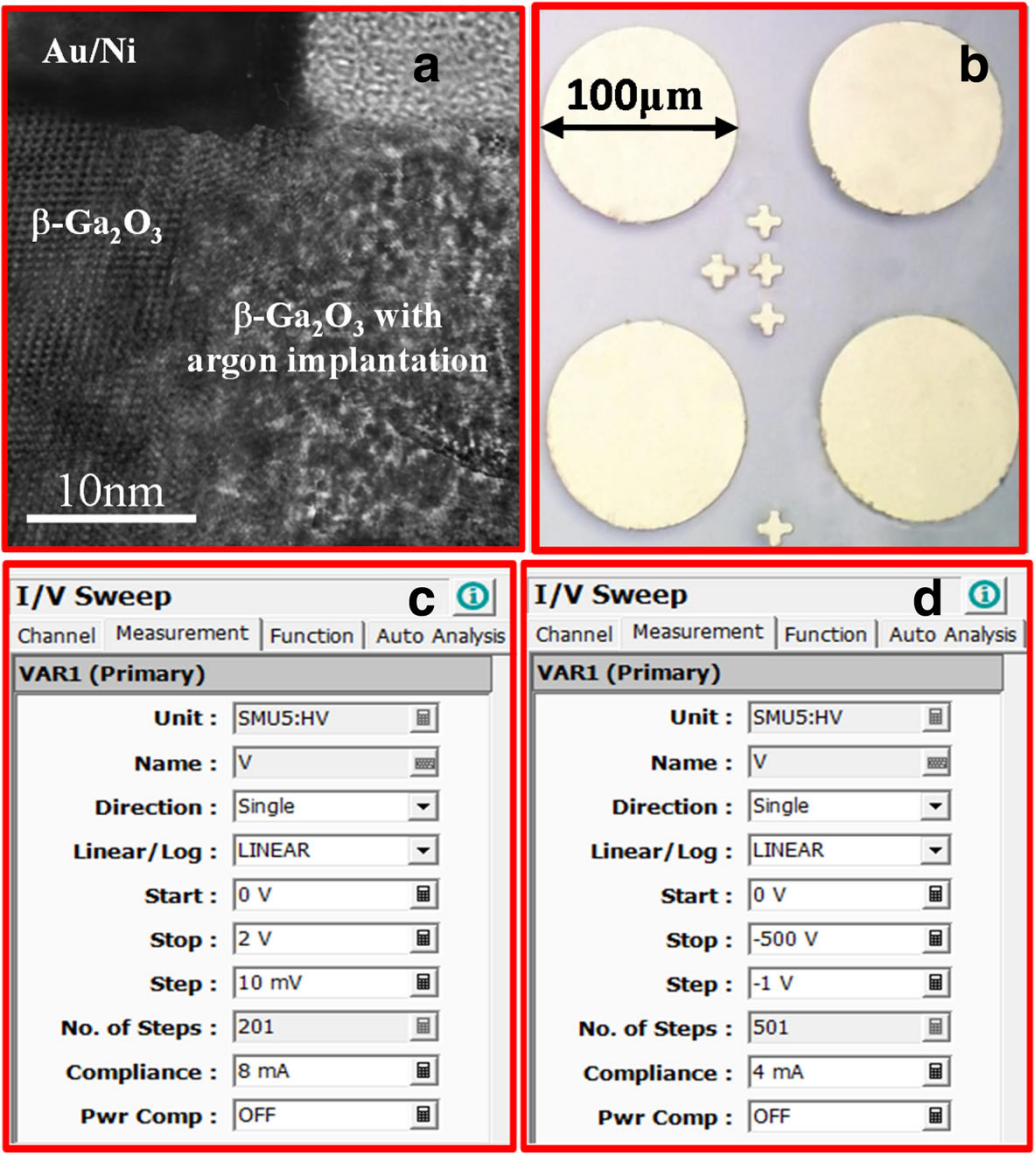
**a** TEM image of sample C and **b** photograph of the terminated β-Ga_2_O_3_ Schottky diode **c** the measurement setup of forward current and **d** reverse current-voltage (I-V) characteristics of the β-Ga_2_O_3_ SBD to obtain the breakdown voltage

The drift layer with the thickness of 10 μm was obtained from high-quality Sn-doped (100)-oriented β-Ga_2_O_3_ bulk substrate by mechanical exfoliation. The β-Ga_2_O_3_ bulk was grown by EFG technique with 4 N pure Ga_2_O_3_ powder as the starting material. Excellent crystal quality and smooth surface were confirmed by high resolution x-ray diffraction (HRXRD) and atomic force microscope (AFM) measurements, as presented in Fig. 1a, b. The full width at half-maximum(FWHM) and root mean square (RMS) were estimated to be 37.4 arcsec and 0.203 nm, respectively. Figure 1c shows the distribution of β-Ga_2_O_3_ substrate sheet resistance with the thickness of 10 μm by a four-point probe measurement. Using carrier concentration of (1.3 ± 0.04) × 10^17^ cm^−3^ and sheet resistance of (563 ± 18.5)Ω/□, the electron mobility is calculated to be 85.3~95.2 cm^2^/Vs by μ_n_ = 1/(R_Sheet_ × *n* × *q* × *t*), where μ_n,_ R_Sheet,_ *n*, *q*, and *t* are the electron mobility, the sheet resistance, the electron concentration, electron charge, and the thickness of β-Ga_2_O_3_ substrate, similar to the reported results [25]. Argon ion implantation with an energy of 50 keV, the dose of 2.5 × 10^14^ cm^−2^, and high temperature annealing at 950 °C for 60 min in N_2_ atmosphere were first performed on the back side, followed by E-beam evaporation of a Ti/Au (20 nm/100 nm) ohmic metal stack and rapid thermal annealing at 600 °C for 60 s in N_2_ ambient. Then the 2-μm-thick photoresist was used as the mask for argon implantation at room temperature with an energy of 50 keV and the dose of 5 × 10^14^ cm^−2^ (sample B) and 1 × 10^16^ cm^−2^ (sample C), respectively. In order to repair the implantation damage and reduce the leakage current under reverse bias, the implanted samples were subjected to a rapid thermal annealing at 400 °C for 60 s under N_2_ ambient [13, 26]. Finally, the circular Schottky anode electrodes with diameter of 100 μm were fabricated on the front side by standard photolithographic patterning, evaporation of Ni/Au (30 nm/200 nm) stack, and lift-off. The reference device without argon implantation was also fabricated (sample A). Figure 2a depicts cross-section TEM of fabricated-Ga_2_O_3_ Schottky diode with argon-implanted edge termination. An almost surface amorphous β-Ga_2_O_3_ layer was created in the implantation region. The actual photograph of the terminated β-Ga_2_O_3_ Schottky diode is shown in Fig. 2b. Figure 2c represents the measurement setup of forward current–voltage (*I*-*V*) characteristics of the β-Ga_2_O_3_ SBD, while the measurement voltage ranges between 0 and 3 V and the step is 10 mV. Figure 2d depicts the measurement setup of reverse current–voltage (*I*-*V*) characteristics of β-Ga_2_O_3_ Schottky rectifiers to obtain the breakdown voltage, while the measurement voltage ranges between 0 and − 500 V and the step is − 1 V.

## Results and discussion

**Fig. 3:**
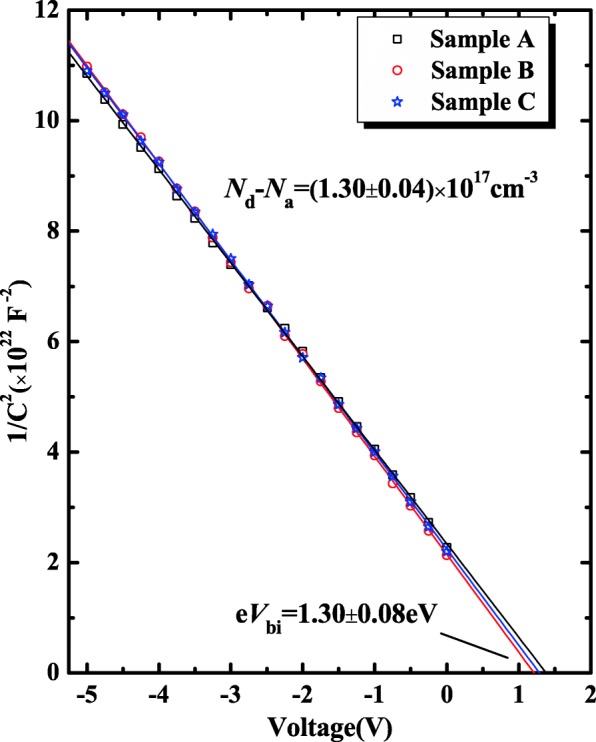
1/*C*^2^-*V* plots of three β-Ga_2_O_3_ SBD samples

Figure 3 shows the experimental 1/*C*^2^ versus *V* characteristics of three SBD samples at room temperature. The effective carrier concentration *N*_*d*_-*N*_*a*_ of β-Ga_2_O_3_ drift layer and built-in potential (*eV*_*bi*_) are extracted to be (1.3 ± 0.04) × 10^17^ cm^−3^ and (1.30 ± 0.08) eV, respectively. According to the following equations, the Schottky barrier height *φ*_b_CV_ is calculated to be (1.32 ± 0.08) eV.

1 $$ \frac{1}{C^2}=\frac{2}{q\varepsilon {A}^2\left({N}_d-{N}_{\mathrm{a}}\right)}\left({V}_{bi}-V\right) $$

2 $$ e{\varphi}_b={eV}_{bi}+\left({E}_c-{E}_f\right)-e\Delta \varphi $$

3 $$ {E}_c-{E}_f= kT\ln \left(\frac{N_c}{N_d-{N}_a}\right) $$

4 $$ e\Delta \varphi ={\left\{\frac{e}{4\pi \varepsilon}{\left[\frac{2{eV}_{bi}\left({N}_d-{N}_a\right)}{\varepsilon}\right]}^{1/2}\right\}}^{1/2} $$

where *A*, *q*, and *ε* are Schottky contact area, electron charge, and permittivity of β-Ga_2_O_3_. *E*_*c*_, *E*_*f*_, *eΔφ*, *k*, *T*, and *N*_*c*_ are the conduction band minimum, Fermi level, potential barrier lowering caused by the image force, Boltzmann constant, absolute temperature in K, and effective density of states of the conduction band, respectively.

**Fig. 4:**
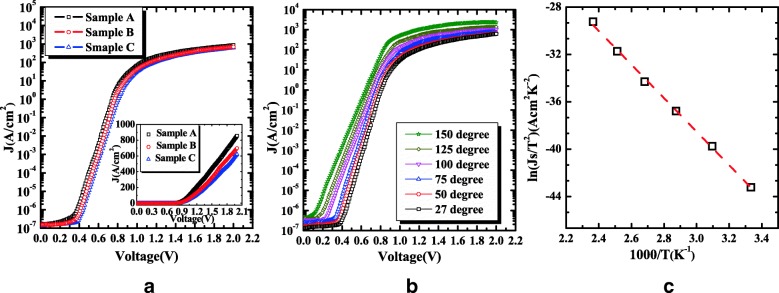
**a** The forward *J*-*V* characteristics of the terminated and unterminated β-Ga_2_O_3_ at room temperature and **b** the temperature-dependent forward *J*-*V* characteristics of sample C from 300 to 423 K. **c** Richardson’s plot of ln(*J*s/*T*^2^) vs 1000/*T* of sample C

Figure 4a represents the forward current density-voltage(*J*-*V*) characteristics of the β-Ga_2_O_3_ SBD. Under the forward bias, the argon implantation has no significant effect on the electrical characteristics. The threshold voltage are determined to be 0.91 V, 0.92 V, and 0.95 V for three samples, the *I*_on_/*I*_off_ ratios are all larger than 10^9^ at room temperature and by fitting the linear region, the specific on-resistances (*R*_on_) are 1.7,2.1 and 3.3 mΩ cm^2^, and forward current densities at 2 V are 857, 699, and 621 A/cm^2^ for three samples, respectively, as shown in Fig. 4a inset. The current densities are higher and the specific on-resistances are lower than or comparable to the reported values for the higher conductivity and carrier density in the drift layer [12, 13, 26–30].

In order to investigate the effects of argon implantation on the temperature dependence of the forward characteristics, the forward *J*-*V* measurements of sample C are conducted from 300 to 423 K, as shown in Fig. 4b. The ideal factor *n* and Schottky barrier height *φ*_*b_JV*_ are determined to be 1.06 and 1.22 eV at room temperature, lower than the *φ*_b_CV_ of (1.32 ± 0.08) eV, according to the thermionic emission (TE) model [31, 32]. With the temperature increasing, the *n* decreases from 1.06 to 1.02 and the barrier height reduces slightly but is almost constant at 1.21 ± 0.01 eV over the temperature range, which is contrary to the barrier height decrease of an ideal Schottky diode with temperature increase but has been observed in fabricated β-Ga_2_O_3_ SBD [26]. Using the equation ln(*J*s/*T*^2^) = ln(A*)-q*ϕ*_b_/*kT*, the barrier height *ϕ*_b_ and the effective Richardson constant A* are determined to be 1.22 eV and 48.5 A/cm^2^ K^2^ for sample C from the slope and the *y*-axis intercept of the linear region of the plot, as shown in Fig. 4c. Furthermore, the extracted A* values for tens of devices on the three samples are between 24 and 58 A/cm^2^ K^2^, consistent with the previous experiment results and theoretical value, 24–58 A/cm^2^ K^2^, with the effective electron mass m^*^ = 0.23–0.34 m_0_ of β-Ga_2_O_3_ [33–37].

**Fig. 5:**
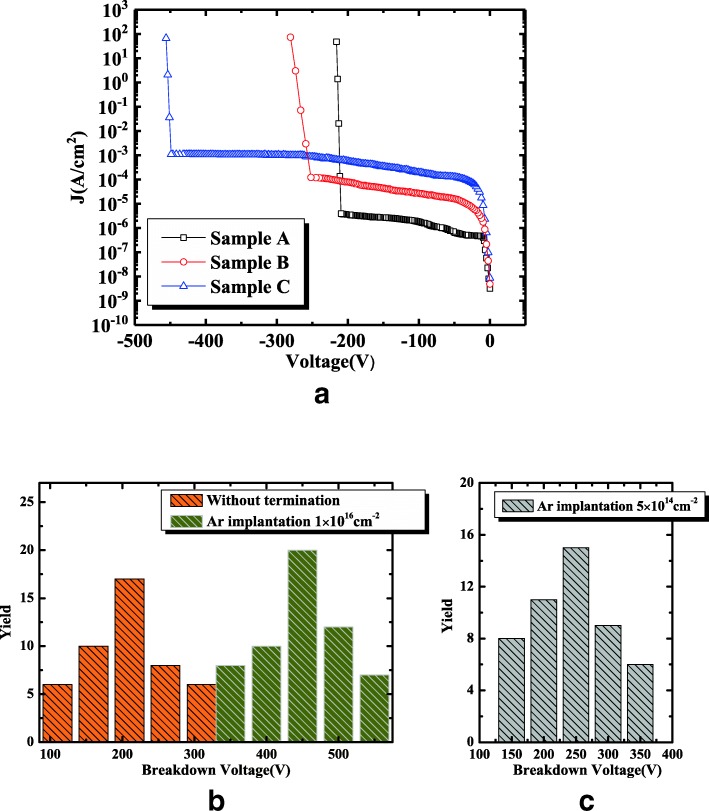
**a** The reverse *J*-*V* characteristics of the β-Ga_2_O_3_ samples at room temperature. **b** and **c** Distribution of breakdown voltages of β-Ga_2_O_3_ SBDs with and without argon implantation

Figure 5a depicts the reverse *J*-*V* characteristics of the samples. After argon implantation, the breakdown voltage increases from 209 to 252 and 451 V and the Baliga figure-of-merit (V_BR_^2^/R_on_) for three samples are approximately 25.7, 30.2, and 61.6 MW cm^−2^, respectively. During implantation, some defects may be introduced and lead to the significant and undesirable increase in leakage current, which was also reported in SiC and GaN Schottky diode devices [18–20]. Although the thermal annealing was conducted after argon implantation, there are still slightly larger leakage currents for samples B and C. Therefore, the investigation detail of the post annealing temperature and duration on the forward and reverse electrical characteristics should be addressed in the following paper.

**Fig. 6:**
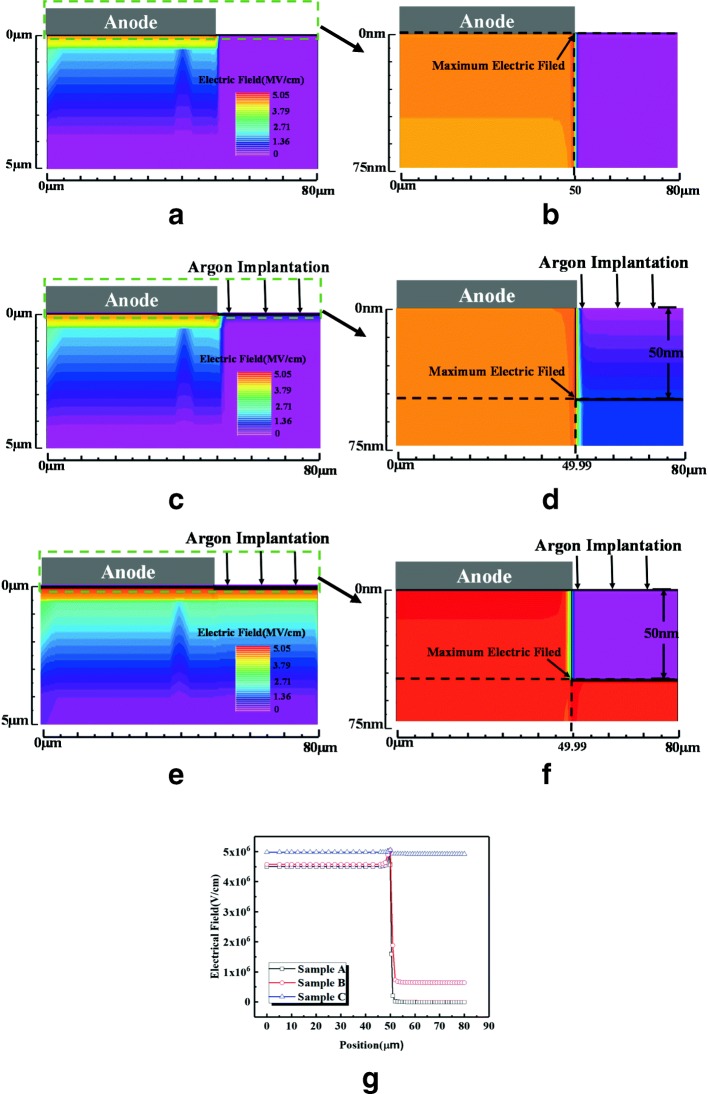
Simulation of the electric field distribution at breakdown voltage of samples A–C (**a**, **c**, **e**), the magnified image of selected regions in dashed box (**b**, **d**, **f**, **g**), the simulated electric field vs the position along the dashed line in (**b**, **d**, **f**) for three samples, Au/Ni/β-Ga_2_O_3_ interface for sample A, 50 nm below the interface for samples B and C, respectively. The maximum field is 5.05 MV/cm

Figure 5b, c presents the distribution of breakdown voltages of β-Ga_2_O_3_ Schottky rectifiers with and without argon implantation. The ideal plane parallel breakdown voltages of these devices are determined as 553 ~598 V, using the critical electrical field of 5.1~5.3 MV/cm [11, 39]. The breakdown voltage without argon implantation is about 110 ~310 V, which is around the 50% of the ideal values. However, with argon-implantation dose of 5 × 10^14^ cm^−2^, the breakdown voltage increases to 150~350 V, not much larger than the reference device, while with the dose of 1 × 10^16^ cm^−2^, the breakdown voltage is approaching the ideal values. In this work, the maximum breakdown voltage of 550 V can be obtained. In addition, the electric field distribution at the breakdown voltage was simulated. For simplification, a single midgap acceptor level was added with the implantation depth of 50 nm determined by the TRIM simulation and the incomplete ionization model was also considered [38], as shown in Fig. 6. Obviously, the high-resistivity layer effectively smooths out electric field at the junction corners and enhances the breakdown voltage greatly in comparison with the reference sample. The maximum electric fields at breakdown voltage are all about 5.05 MV/cm, similar to the reported results [11, 39], while the position changes from the anode corner at the interface to the overlap corner just under the implantation region, as indicated in Fig. 6d, e.

## Conclusions

Vertical Au/Ni/β-Ga_2_O_3_ Schottky barrier diodes with edge termination formed by argon implantation were fabricated on β-Ga_2_O_3_ drift layer mechanically exfoliated from high-quality (100)-oriented β-Ga_2_O_3_ bulk substrate. Compared with the control device, the specific on-resistances (*R*_on_) increases from 1.7 to 2.1 and 3.3 mΩ cm^2^ and the breakdown voltage increases from 209 to 252 and 451 V for implantation dose of 5 × 10^14^ cm^−2^ and 1 × 10^16^ cm^−2^, respectively, with a larger reverse leakage current. The maximum electric field at breakdown voltage is about 5.05 MV/cm, much larger than that of SiC and GaN.

## Abbreviations

AFM: Atomic force microscope
EFG: Edge-defined film-fed growth
FWHM: The full width at half-maximum
FZ: Floating-zone
HRXRD: High resolution x-ray diffraction
JTE: Junction termination extension
MOSFET: Metal-oxide-semiconductor field-effect transistor
RMS: Root mean square
SBD: Schottky barrier diode
TE: Thermionic emission
